# Dorsally Placed Buccal Mucosal Graft Urethroplasty in Treatment of Long Urethral Strictures Using One-Stage Transperineal Approach

**DOI:** 10.1155/2014/792982

**Published:** 2014-07-06

**Authors:** Kamyar Tavakkoli Tabassi, Alireza Ghoreifi

**Affiliations:** Urology Department, Imam Reza Hospital, Mashhad University of Medical Sciences, Mashhad Center for Reconstructive Urology and Plastic Surgery of External Genitalia, P.O. Box 9137913316, Iran

## Abstract

*Objectives*. To evaluate the results of one-stage buccal mucosal urethroplasty in treatment of long urethral strictures. *Methods*. This retrospective study was carried out on 117 patients with long urethral strictures who underwent one-stage transperineal urethroplasty with dorsally placed buccal mucosal grafts (BMG). Success was defined as no need for any intervention during the follow-up period. *Results*. Among 117 patients with mean age of 39.55 ± 15.98 years, the strictures were located in penile urethra in 46 patients (39.32%), bulbar urethra in 33 (28.20%) and were panurethral in 38 (32.48%). The etiology of the urethral stricture was sexually transmitted disease (STD) in 17 (14.53%), lichen sclerosus in 15 (12.82%), trauma in 15 (12.82%), catheterization in 13 (11.11%), transurethral resection (TUR) in 6 (5.13%), and unknown in 51 (43.59%). The mean length of strictures was 9.31 ± 2.46 centimeters. During the mean followup of 18.9 ± 6.7 months success rate was 93.94% in bulbar strictures, 97.83% in penile strictures, and 84.21% in panurethral strictures (*P* value: 0.061). *Conclusions*. The success rate of transperineal urethroplasty with dorsally placed buccal mucosal grafts is equal in different sites of strictures with different etiologies. So reconstruction of long urethral strictures may be safely and effectively performed at a simple single operative procedure using this method of urethroplasty.

## 1. Introduction

Urethral stricture is a relatively common disease in men with different etiologies [1]. There are different treatments for short urethral strictures, including simple dilatation, internal urethrotomy, scar excision, and end-to-end anastomosis, while management of long urethral strictures still remains a great challenge for urologists and there is renewed controversy over the best means of reconstruction [2–4]. The two-stage urethroplasties with or without use of free graft are the conventional techniques used for the treatment of long anterior urethral strictures [2–4]. Although augmented anastomotic techniques are currently suggested for these kinds of strictures, the material for reconstruction (flap or graft) and location of the graft on the urethral surface (ventral or dorsal) has become a contentious issue [4]. We retrospectively evaluated the results of our experience in one-stage transperineal urethroplasty using dorsally placed buccal mucosal graft and its outcomes in the treatment of long urethral strictures.

## 2. Material and Methods

### 2.1. Patient Population

In this retrospective study we evaluated 117 patients who underwent buccal mucosal graft urethroplasty for treatment of long urethral strictures at the Urology Department of Imam Reza Hospital between December 2006 and December 2012. Preoperative studies included retrograde urethrography, voiding cystography, and urethroscopy.

### 2.2. Surgical Method

**Figure 1:**
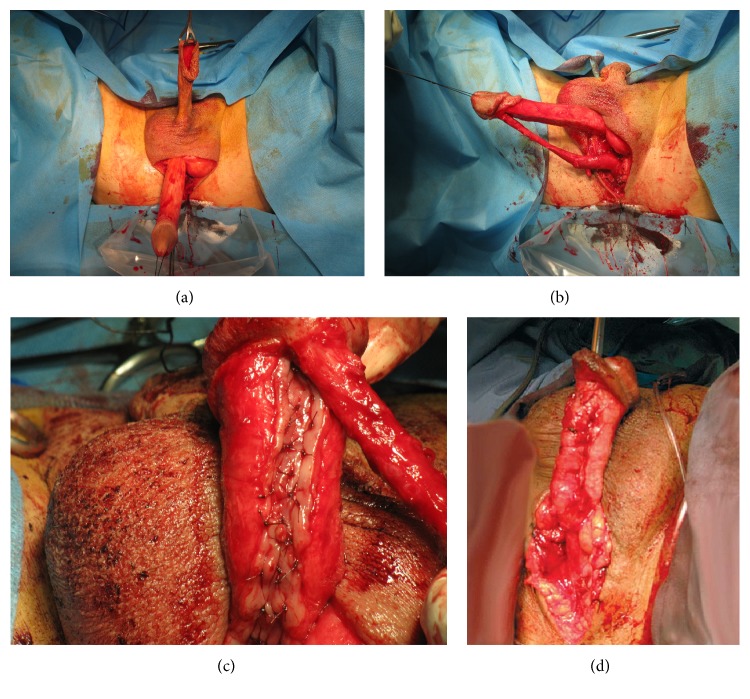
Different steps in surgical procedure.

All of the patients underwent one-stage transperineal repair of long urethral stricture with dorsally placed BMG. In lithotomy position and under general anesthesia a circumcisional incision was made and the penis was degloved. Midline of the perinea was incised and the penis was brought to the perineal incision (Figure 1(a)). The corpus spongiosum, from beginning at the glans of penis to the sphincter, was separated from the corpora cavernosa (Figure 1(b)). A longitudinal incision was made on the dorsal aspect of the urethral stricture. A maximal length of the buccal graft, with about 1.5 to 2 cm width, was harvested from one or two cheeks. After that, grafts were thinned and placed on the dorsal aspect of the urethra and fixed to the tunica albuginea of the corpora cavernosa by applying several sutures using 5–0 vicryl sutures to prevent dead spaces (Figure 1(c)). The urethra was retubularized by suturing the edges of incised urethra to the rims of the buccal graft over an 18F silicon catheter (Figure 1(d)). The penis was replaced in normal anatomy. After placing a drain, the perineum was closed in anatomic layers and the penile skin was placed back in its first position. The patients remained bed rest for 72 hours and were discharged on the 5th to 7th postoperative day.

### 2.3. Followup

**Figure 2:**
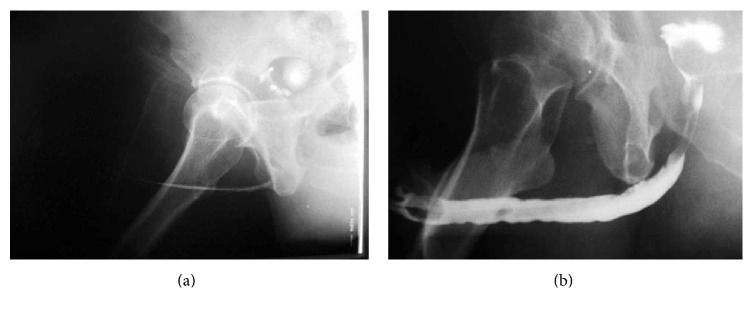
Retrograde urethrography before (a) and after (b) the procedure.

The urethral catheter was kept for 21 days. At the end of the 3rd week, retrograde urethrography was performed (Figure 2). If extravasation was present, then the urethral catheter remained for another 14 days; if not, then the catheter was removed at that time. Follow-up visits were every 3 months for the first year, every 6 months thereafter, and whenever the patients had a problem. During each follow-up visit, careful history taking, physical examination, and urine analysis and culture were performed.

Cystoscopy was done at the end of the third month. If there were symptoms, such as poor urine flow rate, retrograde urethrography was done to rule out a stricture. Failure was defined as a need to any intervention during the follow-up period. We also recorded and evaluated the complications including wound infections, development of meatal stenosis, urethrocutaneous fistula formation, recurrent stricture, erectile dysfunction, penile chordee or deformity, urethral diverticula formation, urinary incontinence or other urinary dysfunctions, lower limb complications due to lithotomy position, and buccal donor site complications. To minimize complications we used different strategies. To avoid wound infections we used intravenous prophylactic antibiotic (cefazolin 1 gr) 30 minutes before operation and continued oral antibiotic while the patient had foley catheter. If wound infections occurred, they were treated with appropriate suppressive antibiotics. To minimize neurologic complications of the lower extremities we decided to change the position of the patients who underwent long time operations (more than 3 hours) for a while during operation from lithotomy to supine. The patients have ambulated 3 days after surgery and physiotherapy exercises have been used while they were on bed rest. All the patients were advised to use antiseptic mouthwashes postoperatively to reduce buccal complications.

### 2.4. Statistical Analysis

The data were analyzed using SPSS software (version 16.0) with chi-square test. *P* values less than 0.05 were considered statistically significant. We analyzed the results including success and complication rates and age, history of previous surgery, and etiology of strictures in three distinct groups according to the site of stricture (penile, bulbar, and panurethral).

## 3. Results

117 patients with mean age of 39.5 ± 16 years underwent buccal mucosal graft urethroplasty in our reconstructive centre. The etiology of the urethral stricture was sexually transmitted disease (STD) in 17 (14.53%), lichen sclerosus in 15 (12.82%), trauma in 15 (12.82%), catheterization in 13 (11.11%), transurethral resection (TUR) in 6 (5.13%) and unknown in 51 (43.59%). The strictures were located in penile urethra in 46 patients (39.32%), bulbar urethra in 33 (28.20%) and were panurethral in 38 (32.48%). The mean length of strictures was 9.31 ± 2.46 centimeters. The previous treatments done for the patients included the following: urethrotomy in 29 (24.79%), dilatation in 24 (20.51%), and urethroplasty in 2 (1.70%), and 62 patients (52.99%) did not receive any treatment before.

With mean follow-up time of 18.9 ± 6.7 months overall success rate was 92.31% and failure rate was 7.69%.

46 patients were involved with penile strictures. The mean length of the stricture was 7.87 ± 2.74 centimeters. The etiology of strictures in this group was lichen sclerosus in 11 (23.91%), catheterization in 10 (21.74%), STD in 5 (10.87%), TUR in 4 (8.70%), and unknown in 16 (34.87%). Success rate was 97.83% and failure rate was 2.17%. Also bulbar strictures were seen in 33 patients. The mean length of the stricture was 5.67 ± 1.53 centimeters. The etiology of strictures in this group was trauma in 7 (21.21%), catheterization in 3 (9.09%), TUR in 2 (6.06%), STD in 2 (6.06%), and unknown in 19 (57.58%). Success rate was 93.94% and failure rate was 6.06%. Finally 38 patients were involved with panurethral strictures. The mean age of patients was 44.5 ± 13.8 years. The mean length of the stricture was 14.39 ± 3.12 centimeters. The etiology of strictures in this group was STD in 10 (26.32%), trauma in 8 (21.05%), lichen sclerosus in 4 (10.53%), and unknown in 16 (42.10%). Success rate was 84.21% and failure rate was 15.79%. So there was no significant difference in success rate between these three groups (*P* value: 0.061). Characteristics of the patients who failed are shown in Table 1.

*Complications.* Overall postoperative complications were wound infection in 10 (8.55%), ring stenosis resulting in urethrotomy in 9 (7.69%), meatal stenosis in 5 (4.27%), and mild chordee in 2 (1.70%). There were no erectile dysfunction, urinary incontinence, and other urinary problems as well as urethral diverticulum postoperatively. Six patients (5.12%) experienced paresthesia in their lower extremities after the operation, which in all cases resolved during the hospitalization period, and were all able to walk normally at the time of discharge. Obturator neuropathy occurred in one patient which was maybe due to compression of obturator nerve during long time lithotomy position, but it was resolved gradually using physiotherapy exercises.

Minor buccal discomfort occurred in 20 patients (17.09%) after surgery but all of them became symptom-free during their followup. We did not meet any major bleeding during the procedure and we did not need any blood transfusion during the procedure and in postoperative period.

## 4. Discussion

Urethral strictures are a frequent source of lower urinary tract disorders in adults. In the developed world today postinflammatory stricture is rare. Iatrogenic causes such as TUR, urethral catheterization, cystoscopy, prostatectomy, brachytherapy, and hypospadias surgery account for about half of the cases of urethral stricture disease treated with urethroplasty. In about 33% of cases no obvious cause can be identified and it may be higher in special locations as Barbagli et al. reported 65.3% of bulbar strictures with unknown etiology [1, 4]. Lichen sclerosus, in which external genitalia (the glans of penis and prepuce) is involved, may be accompanied with long urethral strictures as well [5]. Surgical treatment of urethral stricture diseases is a continually evolving process, and currently there is renewed controversy over the best means of reconstructing the urethra. Moreover, the superiority of one technique over another has not yet been clearly defined [2–4].

Dilation and urethrotomy continue to be the most commonly used techniques, but their failure rates are high with recurrence in 47.6% of patients and many patients progress to surgical repair. Moreover, repeated dilation or urethrotomy exacerbates scar formation, thus adding to stricture length and predisposing to a more difficult definitive open repair and a lower success rate [6, 7]. A recent survey showed that 57.8% of urologists do not perform urethroplasty, whereas 31–33% would continue to manage the stricture by minimally invasive means, despite predictable failure, and most of them believed that the literature supports the use of urethroplasty only after repeated endoscopic failure [8]. In our study about 48% of patients had undergone treatments before and 44% of the failed patients showed history of previous treatments.

Open urethroplasty is the gold standard treatment for urethral strictures, but it is not a routine operation for a general urologist. Since 1993 El-Kasaby et al. reported the first experience with buccal mucosa urethroplasty for treatment of penile and bulbar urethral strictures [9]; buccal mucosa has become an increasingly popular graft tissue for urethral reconstruction. Buccal mucosa is hairless with a thick elastin-rich epithelium and a thin and highly vascular lamina propria; also its use avoids cosmetic disadvantages and consequences caused by the use of genital skin [4]. In 1996, Morey and McAninch fully described the ventral onlay oral mucosal graft urethroplasty and Barbagli et al. described the dorsal free graft urethroplasty [10, 11]. Although Alsikafi et al. in their study showed that there was no significant difference between the use of penile skin graft and buccal mucosal graft for urethral reconstruction, Barbagli et al. showed that the skin graft urethroplasty had a higher failure rate compared to the buccal mucosa graft [12, 13]. Barbagli et al. also showed that the success rate of ventral OMG urethroplasty was 83% in their first study and 91.4% in another study with more followup. On the other hand, the success rate for dorsal type was 85% and 79.2%, respectively. So with the extended followup, the success rate decreased slightly in this group, although this difference may be because it was primarily selected for patients showing complex, long urethral strictures and also recurring after previous urethroplasty [14, 15].

The surgical technique for penile urethral reconstruction is basically selected according to the etiology of the urethral stricture disease. The controversy over the best means of reconstructing the penile urethra has been renewed and, in recent years, free grafts have been making a comeback, with fewer surgeons using genital flaps [16, 17]. Penile urethroplasty using a graft was greatly improved in 1999 when Hayes and Malone suggested an evolution of Snodgrass's longitudinal incision of the urethral plate, laying an oral mucosal graft into the incised urethral plate [18]. After that, Asopa et al. in 2001 popularized a similar technique using ventral sagittal urethrotomy approach for penile stricture repair [19]. In Barbagli's experience using Asopa's technique, oral mucosa was better than skin graft material, but the difference (82% versus 78%) does not justify the use of oral mucosa as a first choice [20]. Also Pisapati et al. reported success rate of 87% using the same technique for recurrent anterior urethral strictures which none of the recurrences had been occurred in penile strictures [21]. The choice of substitute material (oral mucosa versus preputial skin) should be based primarily on surgeon preference and background. The controversy over the best means of reconstructing the penile urethra, using flap or graft, as well as the one-stage or two-stage urethroplasty is still under debate [4]. Traditionally if the penis is generally normal and the penile skin, urethral plate, corpus spongiosum, and dartos fascia are suitable for urethral reconstruction, one-stage urethroplasty is the surgery of choice, but in patients who have experienced failed hypospadias repair or in whom the penile skin, urethral plate, and dartos fascia are not suitable for urethral reconstruction, two-staged urethroplasty is recommended [16].

Although recently a new one-stage technique that involves a deeply longitudinal midline incision of the urethral plate and the suturing of buccal mucosal tissue as an inlay graft into the bed obtained within the urethral plate has been described by different authors, the long-term results in a large series of patients treated with this new one-stage penile graft urethroplasty are not available in the current literature [4]. In our experience on the mean followup of 19.4 ± 6.9 months, success rate for penile urethroplasty using dorsally placed onlay BMG was 97.83%. Postoperative complications seen in this group were wound infection, meatal stenosis, and ring stenosis in 6.25%, 4.35%, and 2.17%, respectively. So our technique may be suitable with minimal complications for penile strictures, even for the patients with lichen sclerosus (23.91% of our patients involved with this disease).

The surgical technique used in the repair of the bulbar urethral stricture is selected mainly according to stricture length. BMG urethroplasty is the most widespread method for the repair of long strictures in the bulbar urethra, but the location of the graft on the urethra surface (dorsal versus ventral) has become a contentious issue. In Barbaglie's experience, the placement of the grafts on the ventral, dorsal, or lateral surface of the bulbar urethra provided the same success rates (83% to 85%) and stricture recurrence was uniformly distributed in all patients [14]. Also other studies reported the same results and in Abouassaly and Angermeier's study final success rate was 92% in mean followup of 29.5 months [22]. Recently, Barbagli et al. reviewed the patterns of failure following bulbar substitution urethroplasty and investigated the prevalence and location of anastomotic fibrous ring strictures occurring at the apical anastomosis between the graft and urethral plate and were uniformly distributed among the different surgical techniques, using either skin or buccal mucosal grafts [23]. In our study at the mean followup of 17.7 ± 7.4 months, success rate was 93.94%; so dorsal onlay buccal mucosal graft urethroplasty may be a successful method for treatment of long bulbar strictures with minimal complications.

Management of panurethral strictures is challenging for urologists and how to treat these patients is still a difficult and controversial issue in the field of reconstructive urethral surgery. In the authors' first experience in which the BMG was placed dorsally in treatment of panurethral strictures the success rate was 88.2%, which was comparable with other methods. Furthermore, complication rate was higher in older aged group, but success rate was the same in older and younger groups of patients [24]. Also Kulkarni et al. reported overall success rate of 83.7% in panurethral urethroplasty. In their study success rate was 86.5% for primary urethroplasty and 61.5% in patients in whom urethroplasty had previously failed [25]. In our study 38 patients had panurethral strictures with mean length of 14.39 ± 3.12 centimeters and STD was the most common known etiology. On the mean followup of 12 ± 5.7 months success rate was 84.21% and failure rate was 15.79%. Although the success rate in these patients was lower than the penile and bulbar group, this difference was not statistically significant (*P* value: 0.061). Also postoperative complications seen in this group were comparable with other two groups. So our technique of urethroplasty may be a successful method in the management of panurethral strictures and its success is relatively comparable with the results of other studies.

The main weakness of our study is that it is retrospective and not prospective. Also, more followup is mandatory to calculate long-term failures.

## 5. Conclusion

Reconstruction of long urethral strictures may be effectively performed in a single operative procedure using a transperineal approach with combinations of dorsal BMG.

The results are comparable to those of published series using the dorsal BMG through the standard dorsal two-stage urethroplasties, in which there is a good and direct exposure of the stricture segment. Also this method may be effective in all types of urethral strictures (penile, bulbar, and panurethral) with relatively acceptable complications.

### 5.1. Take Home Message

Dorsally placed buccal mucosal graft urethroplasty using one-stage transperineal approach is a feasible method in treatment of long urethral strictures with minimal complications.

### 5.2. Lessons Learnt

Urethral stricture is a bothersome disease and all patients who underwent successful urethroplasty experienced a great change in their quality of life.

## Figures and Tables

**Table 1:** Characteristics of patients who failed in operation.

Patient number	Age (year)	Etiology	Location	Length (cm)	Previous treatment	F/U (month)
1	53	Unknown	Bulbar	4	—	12
2	36	LS	Penile	8	Urethrotomy	18
3	68	Unknown	Panurethral	12	—	12
4	51	Unknown	Panurethral	12	—	9
5	53	Unknown	Panurethral	20	Dilatation	24
6	51	Unknown	Panurethral	14	Urethrotomy	12
7	41	Trauma	Panurethral	15	—	24
8	68	Unknown	Panurethral	12	Dilatation	9
9	38	Trauma	Bulbar	6	—	18
